# Human Breathable Air in a Mediterranean Forest: Characterization of Monoterpene Concentrations under the Canopy

**DOI:** 10.3390/ijerph17124391

**Published:** 2020-06-18

**Authors:** Albert Bach, Ana Maria Yáñez-Serrano, Joan Llusià, Iolanda Filella, Roser Maneja, Josep Penuelas

**Affiliations:** 1 Institute of Environmental Science and Technology (ICTA), Autonomous University of Barcelona (UAB), Z Building, ICTA-ICP, Carrer de les columnes, UAB Campus, Bellaterra (Cerdanyola del Vallès), 08193 Barcelona, Spain; 2 Environment and Human Health Laboratory (EH^2^ Lab), Forest Science and Technology Center of Catalonia, Crta. de St. Llorenç de Morunys, km 2, 25280 Solsona, Spain; roser.maneja@uab.cat; 3 CREAF, Campus Universitat Autònoma de Barcelona, Cerdanyola del Vallès, 08193 Barcelona, Catalonia, Spain; a.yanez@creaf.uab.cat (A.M.Y.-S.); j.llusia@creaf.uab.cat (J.L.); iola@creaf.uab.cat (I.F.); josep.penuelas@uab.cat (J.P.); 4 CSIC, Global Ecology Unit CREAF-CSIC-UAB, Bellaterra, 08193 Barcelona, Catalonia, Spain; 5 Forest Science and Technology Center of Catalonia, Crta. de St. Llorenç de Morunys, km 2, 25280 Solsona, Spain; 6 Geography Department, Autonomous University of Barcelona (UAB), B Building, UAB Campus, Bellaterra (Cerdanyola del Vallès), 08193 Barcelona, Spain

**Keywords:** monoterpenes, forest exposure, PTR-MS, forest chemistry, BVOCs, forest bathing

## Abstract

Monoterpenes have been identified as potential determinants of the human health effects induced by forest exposure. The present study characterizes the total monoterpene concentrations at nose height in a Mediterranean Holm oak forest located in North-East Iberian Peninsula during the annual emission peak (summer and autumn: June to November) using a Proton Transfer Reaction–Mass Spectrometry (PTR-MS). Results show a strong variability of the total monoterpene concentrations in season and daytime. The concentration peak appears during July and August. These two months displayed two average maxima in their diel cycles: One during early morning (from 6:00 to 8:00, 0.30 ppbv for July and 0.41 ppbv for August) and another one at early afternoon (from 13:00 to 15:00, 0.27 ppbv during July and 0.32 ppbv during August). Monoterpene concentrations were strongly related with the temperature (exponentially) and solar radiation (rectangular hyperbolic relationship). The concentrations registered here are similar or higher than in previous ex situ studies showcasing the effects of forests on human health. These findings provide relevant data for the scientific and healthcare community by improving the understanding of monoterpene dynamics at nose height and suggesting further research on the effects of forests on human health, particularly in the Mediterranean region.

## 1. Introduction

Interest in understanding if and how the exposure to forests leads to human health benefits is growing among the studies analyzing the effects of nature on human health [1,2,3,4]. However, evidence connecting particular forest variables and health effects is still inconclusive, due to the lack of forest descriptions and the high heterogeneity of approaches and results within the studies performed so far [5]. Furthermore, Mediterranean forests appear to be scarcely studied in comparison with Asian ones [5], where abiotic variables and forest ecosystems may differ. This growing body of research has argued that biogenic volatile organic compounds (BVOCs) may partly explain the health effects induced by forest exposure [6,7,8,9]. BVOCs emitted by forests have diverse roles at multiple scales; from cellular protection and defense at the foliar level, through to chemical signaling at the regional level, up to influencing rainfall at the ecosystems scale [10]. These compounds profoundly affect biosphere–atmosphere interactions by atmospheric reactivity, aerosol growth processes, cloud formation, and therefore radiative balance [11,12].

Monoterpenes are part of the isoprenoid class, the largest class of BVOCs and major components of the forest atmospheres [13]. These compounds are produced by plants as a defensive mechanism against environmental stress and herbivory [13,14,15]. Additionally, monoterpenes are highly reactive compounds that have a high yield in secondary organic aerosol formation [16]. Monoterpene emission vary across plant species and functional type [17,18], which further changes with an altered physiological plant state [19], leading to distinct spatial [20] and temporal [21,22] emissions worldwide. Monoterpenes have been shown to interact with human health in different ways, from stress relief to an effect on the immune function. Inhaling monoterpenes has been shown to decrease blood pressure and cortisol levels [23], improve antibiotics efficiency [24], or boost the immune system [8,25,26], especially by increasing the percentage and activity of natural killer cells [7,9,27,28]. Furthermore, monoterpenes that relevantly affect cellular and animal systems have shown anti-inflammatory, antitumorigenic, or neuroprotective activities [29].

To our knowledge, only one study has analyzed the absorption of monoterpenes in blood after forest exposure, by identifying the monoterpene species present in a coniferous forest atmosphere in serum samples of individuals after walking in the forest [30]. The study demonstrated an increase in the amount of α-pinene in the serum after the individuals walked in the forest as well as differences in monoterpene composition and abundance between coniferous and broad-leaved forest air [30]. In addition, Lee et al. (2018) reported that the mean atmospheric concentration of monoterpenes at a 1.5-m height was higher in a natural than a tended forest [8], suggesting that vegetation characteristics derived from management may affect terpenes’ concentrations under the canopy.

While studies are increasingly providing evidence on the role of monoterpenes in human health, research is needed to describe and predict the composition and abundance of monoterpenes at nose height under forest canopies, analyze the absorption and metabolism of these chemicals by humans, and identify the mechanisms leading to health effects. To address this gap, this article characterized the total monoterpene concentrations at nose height in a Mediterranean holm oak forest during the annual emission peak and posterior months and sheds light on the potential effects of such concentrations on human health.

## 2. Materials and Methods

### 2.1. Measurement Site

**Figure 1:**
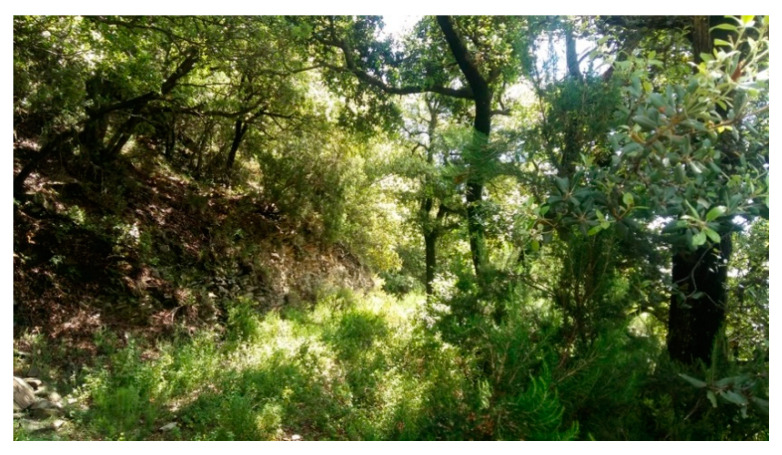
Sampling site.

The study site was located in Montseny Natural Park—Biosphere Reserve (NE Iberian Peninsula, Spain) in a Holm oak forest dominated by *Quercus ilex* (1100 per ha), which is highly representative of the montane holm oak (*Quercus ilex* L.) forests in the northern Mediterranean regions [31], Figure 1. At Montseny, due to coppicing until the 1950s and the posterior selective thinning, holm oaks have nowadays become dense forest of resprout origin [32]. The average diameter at breast height (DBH) for *Quercus ilex* was 16.36 ± 0.917 cm. Other species like *Pinus halepensis* or *Erica arborea* were identified in the study area but not abundantly. Concerning the forest structure, vegetation cover at the 8-m height was 100% while the layers below did not exceed coverages of 10% except for heights of 0.25 and 0.5 m, where the understory was dense and presented coverages of 80%. This forest area has an altitude range from 415 to 550 m.

**Figure 2:**
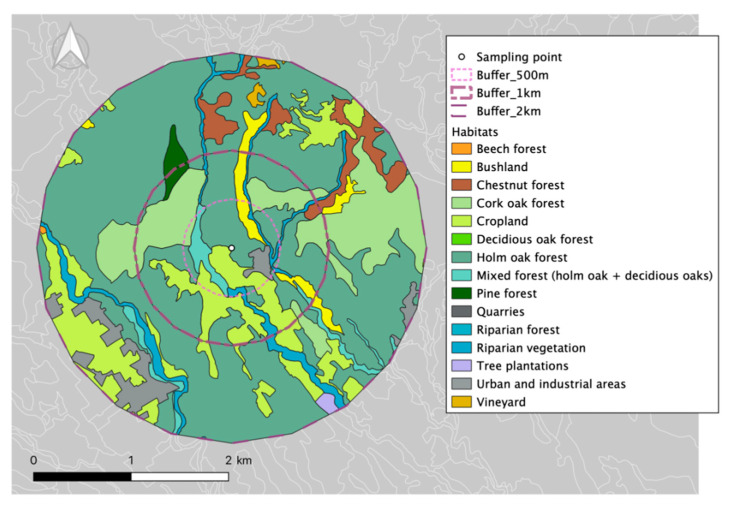
Study area: Sampling point, buffers (500 m, 1 and 2 km), and habitats within the zone.

The habitats within a 500-m radius around our sampling site are presented in Table 1 and illustrated in Figure 2.

### 2.2. Meteorological Conditions

At the sampling site (41°43′43.29″ N,2°26′24.35″ E, 422 m a.s.l.), there was a manual meteorological station with daily values of temperature at 8:00 am, and daily maximum and minimum temperature and precipitation. In order to complement the data with higher frequency datasets, we obtained meteorological data from two other stations of the Servei Meteorològic de Catalunya (i.e., Catalonia’s Meteorological Service): Radiation and humidity from Puig Sesolles station (41°46′25.00″ N, 2°26′15.90″ E, 1668 m a.s.l.) 5 km away from our sampling site, and temperature from Tagamanent station (41°44′51.43″ N, 2°18′10.64″ E, 1030 m a.s.l.) 12 km away from the sampling site. The vapor pressure deficit was calculated with the solar radiation data from Puig Sesolles and the temperature data from Tagamanent.

### 2.3. PTR-MS Sampling Methodology

Measurements of the total monoterpene concentrations were performed with a PTR-MS (Ionicon Analytic GmbH, Innsbruck, Austria) from 26 June to 15 November 2019. One isolated 10-m Teflon tube (OD ¼) was installed at 1.5 m attached with a rope to a holm oak tree. Air was sucked to inside the Park Office where the PTR-MS was located by a pump (JUN-AIR, Benton Harbor, MI, USA). The PTR-MS was operated at standard conditions (2.2 mbar drift pressure, 600 V drift voltage, 127 Td) [33]. A catalytic converter (Supelco Inc., Bellefonte, USA with platinum pellets heated to 380 °C) was used to monitor the background impurities of the PTR-MS. The background signal for each compound was calculated once every hour. Background values were interpolated over the time of the measurements. Humidity-dependent calibrations (using bubbled zero air to dilute the standard, regulated as close as possible to ambient humidity conditions) were performed using a gravimetrically prepared multicomponent standard, including methanol (*m*/*z* 33), acetonitrile (*m*/*z* 42), acetaldehyde (*m*/*z* 45), acetone (*m*/*z* 59), isoprene (*m*/*z* 69), MACR (*m*/*z* 71), MEK (*m*/*z* 73), and α-pinene (*m*/*z* 137), with several dilution steps (Riemer Environmental Inc., Miami, USA).

The PTR-MS technique separates per mass and not per compound, so other compounds than monoterpenes with the same mass might be measured together. However, sporadic GC-MS screening at the site confirmed the presence of monoterpenes (i.e., *m*/*z* 137) in ambient air, more specifically α-pinene and β-pinene.

### 2.4. Data Analysis

Igor Pro (Wavemetrics Inc., Portand, OR, USA) was used for the calculations of BVOCs concentrations, data time series treatment, graphing, and descriptive statistics. We conducted a generalized linear model (GLM) to determine the effect of the available atmospheric data (temperature, precipitation, relative humidity, solar radiation, ozone, and vapor pressure deficit) on monoterpene concentrations using R (Version 1.2.5033). Monoterpene concentrations were log-transformed to ensure normality of the residuals. A *p* < 0.05 was considered significant.

## 3. Results

**Figure 3:**
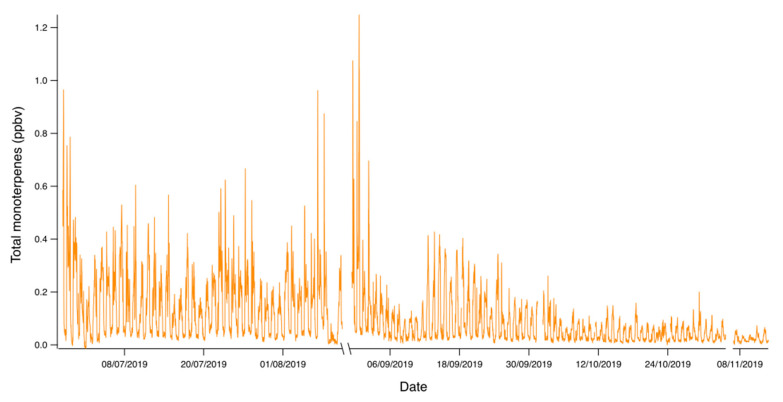
Total monoterpene air concentrations during the sampling period (from June to November 2019). The gaps are due to instrument lack of power in the sampling site.

Figure 3 shows the complete time series of the total monoterpene concentrations measured in this study from June to November 2019, which ranged from 0.02 to 0.13 ppbv (25 percentile and 75 percentile), with maximum values reaching 1.24 ppbv. Total monoterpenes had a clear seasonality with higher concentrations during summer as well as a strong daily pattern with highest concentrations during daylight.

**Figure 4:**
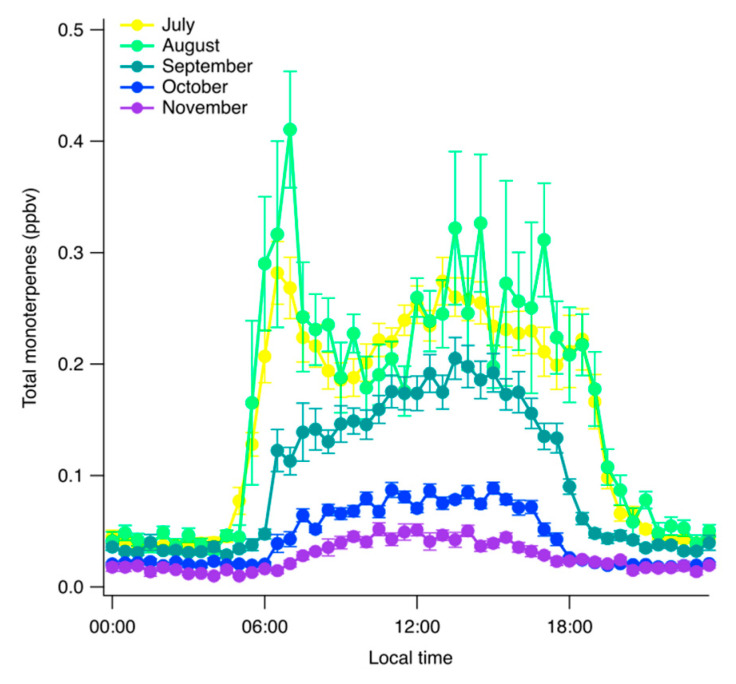
Half-hour average diel cycle of total monoterpene concentrations per month. Error bar represents the standard error.

The average monthly diel cycles (Figure 4) showed that August and July had the highest concentrations. These two months displayed two average maxima in their diel cycles, one during early morning (from 6:00 to 8:00, 0.30 ppbv for July and 0.41 ppbv for August) and another one at early afternoon (from 13:00 to 15:00, 0.27 ppbv during July and 0.32 ppbv during August). These peaks were not identified in the rest of the sampled months (September, October, and November), where total monoterpene concentrations increased with the solar radiation and a peak around 14:00, coinciding with the temperature diel cycle peak. Additionally, concentrations values stayed relatively constant during night, with 0.05 ppbv for July and August decreasing to 0.02 ppbv in November.

**Figure 5:**
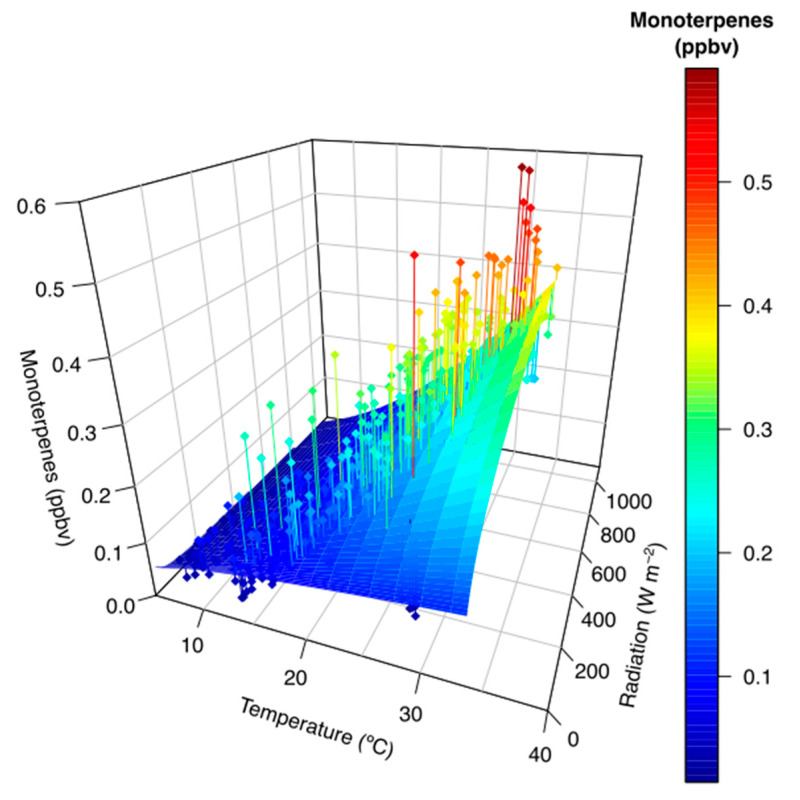
3-D relationship of monoterpene concentrations with temperature and radiation.

Additionally, Figure 5 shows the monoterpene concentrations were strongly related with temperature (exponentially) and solar radiation (rectangular hyperbolic relationship). The evolution of the meteorological variables correlated with the monoterpenes is presented in Figure S1. The GLM analysis demonstrated a significant effect of solar radiation (*t*-value = 22.770; *p*-value = 2 × 10^−16^), temperature (*t*-value = 21.645; *p*-value = 2 × 10^−16^), humidity (*t*-value = 9.506; *p*-value = 2 × 10^−16^), and vapor pressure deficit (vpd) (*t*-value = 6.236; *p*-value = 4.82 × 10^−16^) (Figures S2 and S3 and Table S1).

## 4. Discussion

The present study analyzed the under-canopy variations of monoterpene concentrations in a Mediterranean holm oak forest in Montseny Natural Park—Biosphere Reserve (NE Iberian Peninsula) continuously from June to November 2019 using a PTR-MS. This is the longest time series of total monoterpene concentrations ever measured in a Mediterranean holm oak forest at the nose level. During the sampled period, we identified seasonal and daily patterns. The highest concentrations occurred during summer, and diel cycles changed among months, with peaks in early morning in summer and peaks at midday in the entire period. The total monoterpene variations were significantly linked to abiotic/atmospheric variables, such as temperature and solar radiation, as well as to humidity and vapor pressure deficit (vpd).

The highest monoterpene concentrations occurred during July and August. These results are consistent with other studies that found higher monoterpene concentrations above the canopy level in summer in an holm oak forest (*Quercus ilex* L.) from the same Mediterranean region [34,35]. Previous studies in other regions have as well observed higher monoterpene emissions during summer period [36,37]. Furthermore, monoterpene emission rates measured at the leaf or branch level have proved to be higher from July to August in *Quercus ilex* [34,35,38,39]. This seasonality of monoterpene concentrations is associated to the seasonality of plant monoterpene emission [19,40] mostly linked to the seasonal changes in temperature [41,42] and other meteorological variables, such as solar radiation [17,43,44], which explains as well the decrease during the autumn months (October and November).

We found higher monoterpene concentrations during daytime, with maxima at early morning (from 6:00 to 8:00) and at early afternoon (from 13:00 to 15:00). This early morning peak has been previously reported at a nearby site above forest canopies [38,45,46]. This peak has been attributed to the biogenic monoterpene emissions at the site, which have not yet been influenced by mountain breeze (which starts a few hours later in the day) [46]. The peak at around 14:00 is also related to the biogenic origin of monoterpenes, which is enhanced at maximum temperature and solar radiation. In fact, the maximum at 14:00 coinciding with the temperature peak has been reported previously for areas with light- and temperature-dependent monoterpene-emitting trees, such as tropical trees [47,48]. This differs from other forest sites, including Mediterranean, that documented the diel cycle of the monoterpene concentration to peak at nighttime [37,49,50]. This inconsistency is due to the fact that holm oaks emit monoterpenes in a light- and temperature-dependent manner (so called de *novo* biosynthesis) and not from storage pools at night, like boreal coniferous species [17,43], and thus maximum emissions occur during the middle of the day [51].

Monoterpene emissions are well-known to be affected by atmospheric variables, such as air temperature, solar radiation, and air humidity [17,43,44]. Our results from the GLM analysis support this fact by showing a strong effect of temperature, solar radiation, humidity, and vpd on monoterpene concentrations. Ambient concentrations of a volatile organic compound can be affected by the strength of emissions (either biogenic or anthropogenic), the effectiveness of air mixing, and the strength of sink processes, including deposition and chemical degradation. However, the strong effect of temperature and solar radiation on the observed concentrations suggests the dominance of a biogenic source for total monoterpenes. Further on, as the site is dominated by *Quercus ilex* forests, a dominant monoterpene emitter tree species, we assume that the main origin for the observed concentrations comes from the monoterpene emission of this tree species. This further supports the biogenic origin of the measured concentrations, similar to a previous study at a nearby site [46]. Furthermore, the daily patterns of the concentrations could as well be partly associated to atmospheric vertical stability and wind speed and direction as shown in previous studies [52]. Reasonable interpolation from existing weather stations in this regard could not be provided in the present study, but further research is warranted at this regard.

Our results report similar monoterpene concentrations to another study that has measured these compounds under coniferous forest canopies in Japan [53]. The most abundant monoterpenes in Mediterranean holm oak forests are α-pinene, sabinene, limonene, camphene, and α-phellandrene [38]. Some of these monoterpene species have been previously found to be absorbed by humans after a 60-min walk through a coniferous forest with a six-fold increase of the α-pinene peak in blood after forest exposure [30]. These monoterpenes have been related with physiological changes in humans, in particular α-phellandrene has been significantly associated with an acute insulin reaction [8]. Some ex situ and in vitro studies have shown enhancing effects of monoterpenes on the human immune system but at higher monoterpene concentrations than those we registered in our study (approximately from 50 to 1900 ppbv) [7,9]. Nevertheless, the concentrations we report here (from 114 to 725 ngm^−3^ during the whole sampling period and up to 2230 ngm^−3^ in August) happen to be comparable [53] or much greater [27,28] than in previous in situ studies that identified similar health outcomes to the ones observed in the in vitro and ex situ experiments. The concentrations of monoterpenes of our studied Mediterranean forest are also higher than other in situ studies characterizing the forest atmosphere under the canopy [54] or testing the effects of forests on other human systems and functions [8]. Taken together, these findings suggest that the Mediterranean holm oak forests constitute a suitable forest environment to develop further research on the effects of monoterpenes on human health.

## 5. Conclusions

This study analyzed the monoterpene concentrations at nose height in a Mediterranean Holm oak forest from June to November. We identified a strong variability of the total monoterpene concentrations in season and daytime with its peak during summer. Additionally, the monthly average diel cycles showed two main peaks at early morning (from 6:00 to 8:00) and early afternoon (from 13:00 to 18:00) during July and August. Monoterpene concentrations significantly correlated with air temperature, solar radiation, air humidity, and vapor pressure deficit (vpd).

If BVOCS play a key role in the interaction between forest and human health as highlighted in the literature, our analysis provided relevant information concerning the availability of these compounds at the nose level, thus suggesting that humans walking in the studied forest may be subject to a potentially high absorption of monoterpenes into their bloodstream, especially at early morning and from midday. Our findings contribute to improving the understanding of monoterpene dynamics under the canopy and enhancing the development of more applied research on the effects of forests on human health, particularly in the Mediterranean region.

## Figures and Tables

**Table 1:** Habitat type, surface, and percentages of the total surface per habitat within a 500-m radius from the sampling point.

Habitat	Surface (ha)	Percentage (%)
Holm Oak Forest	33.63	43.50
Cropland	16.83	21.77
Mixed forest (holm oak + deciduous oaks)	9.97	12.90
Urban and industrial areas	5.41	6.99
Bushland	4.94	6.39
Riparian vegetation	4.19	5.42
Cork oak forest	2.34	3.03
TOTAL	77.31	

## Supplementary Materials

The following are available online at https://www.mdpi.com/1660-4601/17/12/4391/s1, Figure S1: Evolution of the meteorological variables during the sampling period; Figure S2: Correlation between air humidity and monoterpenes concentrations; Figure S3: Correlation between air vapor pressure deficit (vpd) and monoterpenes concentrations; and Table S1: Outcomes from the GLM conducted to assess the effect of air temperature, solar radiation, air humidity and vapor pressure deficit (vpd) on monoterpenes concentrations.
