# Supplementary material for: Human Breathable Air in a Mediterranean Forest: Characterization of Monoterpene Concentrations under the Canopy
Source: Int J Environ Res Public Health. 2020 Jun 18;17(12):4391. doi: 10.3390/ijerph17124391 (PMC7344780; doi:10.3390/ijerph17124391)
Supplement: Supplementary file 1 [file ijerph-17-04391-s001.pdf]

Supplementary material

Figure S1. Evolution of the meteorological variables during the sampling period.

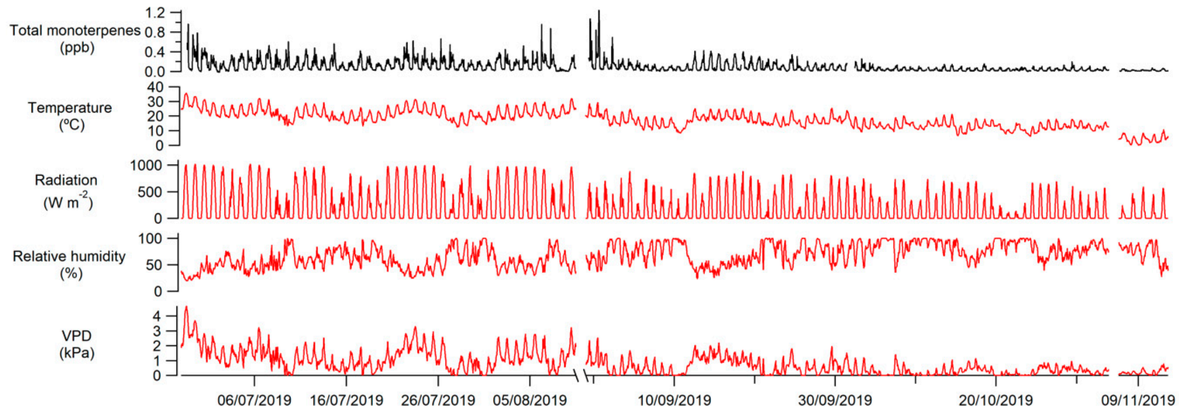

Figure S2. Correlation between air humidity and monoterpenes concentrations.

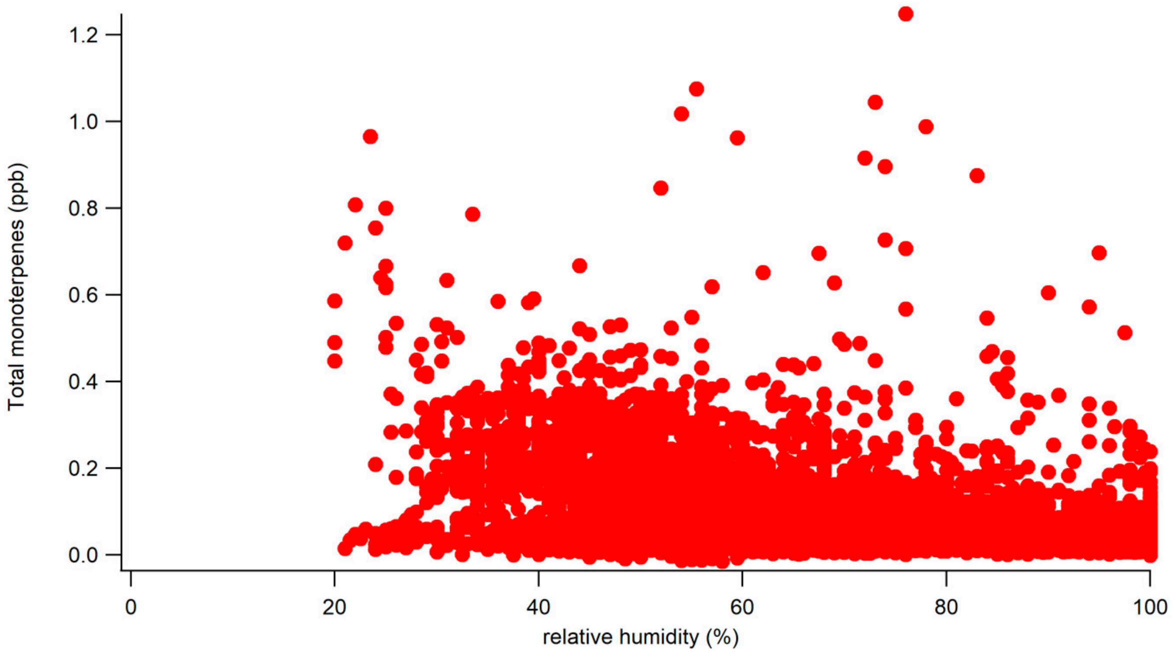

Figure S3. Correlation between air vapor pressure deficit (vpd) and monoterpenes concentrations.

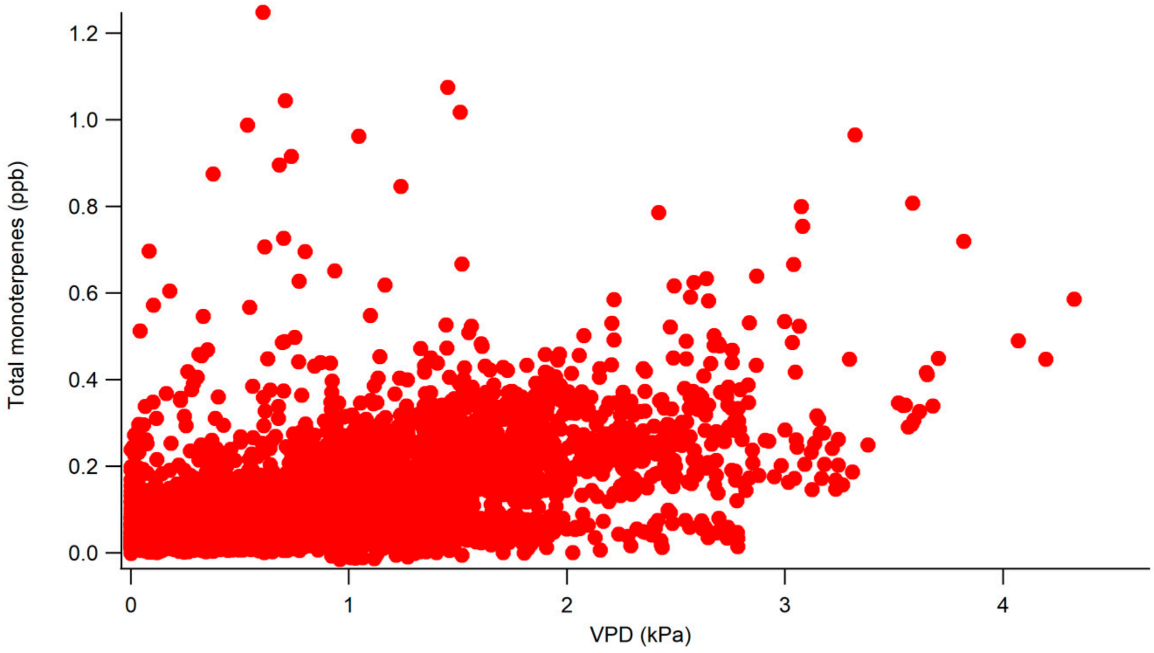

Table S1. Outcomes from the GLM conducted to assess the effect of air temperature, solar radiation, air humidity and vapor pressure deficit (vpd) on monoterpenes concentrations. Please note that a log conversion was applied to the monoterpenes concentrations. Thus, the estimates can not be interpreted straight forward.

	**Estimate**	**Std. Error**	***t* Value**	***p* Value**
Temperature	0.0317485	0.0014668	21.645	2,00E-16
Solar radiation	0.0141676	0.0006222	22.770	2,00E-16
air humidity	0.0073928	0.0007777	9.506	2,00E-16
vpd	0.0055614	0.0008918	6.236	4.82e-10
